# Reducing Stress and Preventing Depression (RESPOND): Randomized Controlled Trial of Web-Based Rumination-Focused Cognitive Behavioral Therapy for High-Ruminating University Students

**DOI:** 10.2196/11349

**Published:** 2019-05-13

**Authors:** Lorna Cook, Mohammod Mostazir, Edward Watkins

**Affiliations:** 1 SMART Lab, Mood Disorders Centre School of Psychology University of Exeter Exeter United Kingdom; 2 College of Life and Environmental Sciences (CLES) School of Psychology University of Exeter Exeter United Kingdom

**Keywords:** cognitive behavioral therapy, depression, prevention, rumination, cognitive, stress, psychological, student health services

## Abstract

**Background:**

Prevention of depression is a priority to reduce its global disease burden. Targeting specific risk factors, such as rumination, may improve prevention. Rumination-focused Cognitive Behavioral Therapy (RFCBT) was developed to specifically target depressive rumination.

**Objective:**

The primary objective of this study was to test whether guided Web-based RFCBT (i-RFCBT) would prevent the incidence of major depression relative to usual care in UK university students. The secondary objective was to test the feasibility and estimated effect sizes of unguided i-RFCBT.

**Methods:**

To address the primary objective, a phase III randomized controlled trial was designed and powered to compare high risk university students (N=235), selected with elevated worry/rumination, recruited via an open access website in response to circulars within universities and internet advertisements, randomized to receive either guided i-RFCBT (interactive Web-based RFCBT, supported by asynchronous written Web-based support from qualified therapists) or usual care control. To address the secondary objective, participants were also randomized to an adjunct arm of unguided (self-administered) i-RFCBT. The primary outcome was the onset of a major depressive episode over 15 months, assessed with structured diagnostic interviews at 3 (postintervention), 6, and 15 months post randomization, conducted by telephone, blind to the condition. Secondary outcomes of symptoms of depression and anxiety and levels of worry and rumination were self-assessed through questionnaires at baseline and the same follow-up intervals.

**Results:**

Participants were randomized to guided i-RFCBT (n=82), unguided i-RFCBT (n=76), or usual care (n=77). Guided i-RFCBT reduced the risk of depression by 34% relative to usual care (hazard ratio [HR] 0.66, 95% CI 0.35 to 1.25; *P*=.20). Participants with higher levels of baseline stress benefited most from the intervention (HR 0.43, 95% CI 0.21 to 0.87; *P*=.02). Significant improvements in rumination, worry, and depressive symptoms were found in the short-to-medium term. Of the 6 modules, guided participants completed a mean of 3.46 modules (SD 2.25), with 46% (38/82) being compliant (completing ≥4 modules). Similar effect sizes and compliance rates were found for unguided i-RFCBT.

**Conclusions:**

Guided i-RFCBT can reduce the onset of depression in high-risk young people reporting high levels of worry/rumination and stress. The feasibility study argues for formally testing unguided i-RFCBT for prevention: if the observed effect sizes are robustly replicated in a phase III trial, it has potential as a scalable prevention intervention.

**Trial Registration:**

ISRCTN Registry ISRCTN12683436; https://www.isrctn.com/ISRCTN12683436 (Archived by WebCite at http://www.webcitation.org/77fqycyBX)

**International Registered Report Identifier (IRRID):**

RR2-10.1186/s13063-015-1128-9

## Introduction

### Background

Depression is the leading cause of disease burden worldwide, accounting for 7.5% of all years lived with disability in 2015 [1], with considerable individual, societal, and economic consequences. Although there are effective evidence-based acute treatments, their impact is limited because of poor access to treatments [2], high rates of nonresponse [3], and the recurrent nature of depression, with 50% to 80% of patients experiencing 2 or more episodes [4]. It is estimated that even with optimal acute treatment at full coverage, only 34% of the disease burden would be averted [5]. As a consequence, a strong case has been made that prevention is needed to reduce the global burden of depression [6].

Preventive interventions, largely using cognitive behavioral therapy (CBT) approaches, can reduce symptoms of depression and prevent the incidence of depression [7-9], with an average reduction of 21% in incidence rates [10]. These meta-analyses suggest that targeted interventions (selective interventions aimed at subgroups with known risk factors and indicated interventions aimed at those with subclinical symptoms) produce larger and longer-lasting effects than universal interventions aimed at entire populations. A meta-analysis [11] of 21 preventive interventions (15 using CBT approaches) found that selective interventions and indicated interventions had lower incidence rate ratios (IRR; 0.72 and 0.76, respectively) relative to controls than universal interventions (IRR=0.90). Merry et al [8] also found that both universal and targeted interventions reduced incidence relative to no intervention in the short-to-medium term (3 to 9 months postintervention) but only targeted interventions reduced incidence at 12 months. Thus, targeting at-risk groups may improve the efficacy of preventive interventions for depression [6], in part because the base rate is higher in targeted samples, so it is easier to detect a significant effect with smaller sample sizes [12].

The incidence of depression rises steeply from the age of 14 years through young adulthood, with increased rates in females (2:1, female:male) emerging at around the age of 12 years and continuing into young adulthood [13]. The UK Adult Psychiatric Morbidity Survey found increasing rates of common mental health disorders (CMDs; incorporating depression and anxiety disorders) among young women (aged 16 to 24 years), rising from 22.2% in 2007 to 28.2% in 2014 [14], with rates in young women almost three times those of young men (10.0%) in 2014. As early onset is linked to greater chronicity [15] and other negative long-term outcomes, such as poor academic and occupational performance [1,16], prevention may be particularly effective and impactful for this age group.

Within this age range, university students are a particularly high-risk group, with a weighted mean prevalence for depression of 30.6% (range 10% to 85%) across 24 studies [17] relative to estimates of 10.8% to 22% in nonstudents of the same age range [18,19]. This increased prevalence may be due to the specific pressures of university and associated lifestyle changes, such as leaving the family home for the first time, forming new friendships, more self-directed learning, and irregular sleep patterns [20]. Students who experience mental health difficulties during their studies are at greater risk of poor academic outcomes [16] and dropout [21].

Despite these challenges, students often do not seek help from relevant services [22,23]. Alternative delivery modes, such as Web-based interventions, offer advantages that may be attractive to students, including availability at any time and place, anonymity which may reduce the stigma of seeking help, and more time to reflect on the treatment material [12,24,25]. A recent systematic review and meta-analysis of 17 Web-based and computer-delivered interventions for higher education students found reductions in depression, anxiety, and stress when compared with inactive controls [26]. However, sample sizes were generally small, and the authors recommend further larger-scale trials to assess the effectiveness of Web-based interventions in university students.

One such relatively large-scale trial by Topper et al [27] tested a guided Web-based targeted preventive intervention for 251 high school and university students aged 15 to 22 years with high levels of self-reported worry and rumination. Participants were randomized to face-to-face group rumination-focused cognitive-behavioral therapy (RFCBT), guided Web-based RFCBT (i-RFCBT), or a no-intervention control group. This preventive intervention is based on RFCBT, previously shown to be effective in treating residual depression [28]. There is considerable evidence that rumination plays a causal role in the onset and duration of major depressive episodes (MDEs) [29,30]. Within a student population, rumination predicts change in depression over 6 months [31]. Rumination interacts with other risk factors to both maintain depression (the combination of rumination, low self-esteem, and stressful life events predicts maintenance of depressive symptoms over 6 weeks [32]) and predict the onset of depressive symptoms (engaging in rumination in response to stress prospectively predicted an increase in subsequent depressive symptoms [33]). These studies suggest that specifically targeting ruminative responses to stressful events could reduce depression.

RFCBT specifically targets repetitive negative thought (RNT), incorporating both rumination and worry, defined as a thinking style that: (1) is repetitive, intrusive, and difficult to disengage from; (2) is perceived as unproductive; and (3) captures mental capacity [34]. RNT is a transdiagnostic process, involved in the onset and maintenance of a range of emotional disorders including depression and anxiety as well as physical health issues [29,35], including in children and adolescents [36-39]. Targeting transdiagnostic risk factors has the potential to improve the efficacy of prevention by impacting on multiple disorders with a single intervention [27].

In the Topper et al [27] trial, both Web-based and group-delivered RFCBT reduced symptoms of depression and anxiety (*d*=0.36 to 0.72), relative to controls. Cumulative incidence rates at the final 12-month follow-up were significantly lower in both RFCBT intervention conditions for depression (14.7% Web-based; 15.3% group) relative to the usual care control condition (32.4% depression), with no difference between i-RFCBT versus group RFCBT. In support of the hypothesized mechanism of change, reductions in worry and rumination were found to mediate the effects of the interventions on prevalence of depression and Generalized Anxiety Disorder (GAD). These findings suggest that targeting rumination may have preventive effects for depression and are consistent with evidence that targeted prevention can be effective in adolescents and young adults.

### Objectives

Topper et al [27] included both secondary and university students to form a heterogeneous sample. Given the evidence that undergraduates may form a distinct at-risk subgroup for depression, the primary aim of this phase III efficacy trial was to test whether these beneficial effects of guided i-RFCBT on onset of depression relative to usual care [27] could be extended to a selective UK high-risk undergraduate population.

We also aimed to address several key limitations of the Topper et al [27] trial: (1) there was no diagnostic interview to assess depression, and self-report measures were only able to estimate point prevalence caseness and (2) as history of depression was not assessed, participants’ previous history of depression was not known, and therefore it was not possible to discriminate whether the intervention prevented first onset or relapse/recurrence of depression. To address these methodological limitations, we included a well-validated diagnostic interview (Structured Clinical Interview for DSM-IV [SCID-I] [40]) to increase accuracy of the current diagnostic status and measure retrospective incidence.

We hypothesized that in high-ruminating undergraduates, guided i-RFCBT, relative to usual care, would significantly reduce the onset of MDEs over the course of the 15 months post randomization follow-up (primary outcome). Rumination has also been found to increase the negative effect of stressful life events on depressive symptoms in young people and students [32,33]. This observed interaction is consistent with the evidence that rumination contributes to depression by exacerbating existing negative mood and negative cognitions and by repetitively dwelling on difficulties [29,30], such as results from stressful events. As such, a tendency to ruminate would be expected to have less impact when things are going well and there is less to dwell on, relative to when things are difficult. We therefore hypothesized that i-RFCBT would be particularly beneficial for high-ruminating undergraduates who were also experiencing stressful life events, as this would be the group for whom rumination would be most detrimental.

As a secondary aim, we explored the feasibility and acceptability of an unguided version of i-RFCBT to prevent depression. Topper et al [27] used i-RFCBT that was guided and supported by a therapist because past evidence suggested that, at least for acute treatment for depression, guided Web-based cognitive behavioral therapy (i-CBT) is significantly more effective than unguided (ie, self-help) i-CBT [41-43], and only guided i-CBT produces similar treatment effects to face-to-face therapy in patients with acute depressive symptoms [24]. A key rationale for Web-based therapy is to increase the coverage, availability, and accessibility of treatment, by potentially reaching large numbers of people through the internet and by overcoming hurdles such as geographical distance, poor mobility, and scheduling appointments during standard office hours. However, any form of guided i-CBT (including i-RFCBT) is necessarily limited in its scalability because coverage is determined by the number and availability of therapists. In contrast, an unguided form of Web-based therapy has nearly limitless scalability as there are no such constraints and, thus, even with smaller effect sizes than guided interventions, has significant potential to reduce the disease burden of depression [44]. Such an intervention would be particularly beneficial for preventing depression because effective prevention requires an intervention to be highly scalable and able to reach very high numbers of people. As a secondary question, we therefore explored the feasibility and acceptability of an unguided version of i-RFCBT in a quasi-phase II pilot arm and estimated its effect sizes to inform a fully powered trial, with regard to incidence rates and symptom levels (descriptives and CIs).

## Methods

### Trial Design

#### Phase III Efficacy Study

The phase III study consisted of a single (researcher) blind parallel-group randomized controlled trial (RCT), comparing guided i-RFCBT versus a usual care control group. For full details, see the trial protocol paper [45] and Current Controlled Trials ISRCTN12683436.

#### Quasi-Phase II Pilot Arm

To assess the feasibility of unguided i-RFCBT, a separate adjunct arm of unguided i-RFCBT was included as a quasi-phase II pilot arm. For efficiency, participants were randomized to this arm within the overall trial design, but there was no direct comparison between the unguided and guided arms. The unguided arm was compared with the control group to estimate the effect sizes of an unguided version of i-RFCBT for the planning of future efficacy trials.

### Participants

Participants were university students resident in the United Kingdom, aged 18 to 24 years, with elevated RNT, defined as scoring above the 75th percentile on at least one measure of worry/rumination: ≥50 on the Penn State Worry Questionnaire (PSWQ; [46]); ≥40 on the Ruminative Response Scale (RRS; [47]), using the same criteria as Topper et al [27]. As a prevention study, participants were excluded if they met the diagnostic criteria for a current (within the past month) MDE. In addition, potential participants were excluded if they reported any of the following: current and significant substance abuse or dependence; current symptoms/diagnosis of psychosis or bipolar disorder; and current psychological therapy or active suicide risk. In line with standard practice, receipt of antidepressant medication was not an exclusion criterion, providing the dose had been stable for at least 1 month.

### Sample Size Calculation and Recruitment

For the primary question comparing guided i-RFCBT to usual care control, assuming a similar hazard ratio of 0.41 for the guided i-RFCBT versus usual care control [27], 75 participants per arm would provide 0.86 power (2-tailed 5% alpha level) to detect this effect. For change in depressive symptoms, the observed effect size was *d*=0.51 [27]. A total of 78 participants per arm would be needed for 80% power to detect a similar effect at the 2-tailed 5% alpha level, allowing for 20% follow-up dropout attrition. With no previous evidence for unguided i-RFCBT, the comparison of unguided i-RFCBT to usual care was conducted as a feasibility study as a first step to conducting a fully powered trial of unguided i-RFCBT. As such, no power or sample size calculations were conducted for the unguided arm. We aimed to recruit the same number (n=78) as the other 2 arms.

The full recruitment procedure is outlined in Cook and Watkins [45]. Briefly, 1834 university departments in the United Kingdom were contacted between November 14, 2013, and December 10, 2014 (1527 contacted twice) and asked to advertise the study. In total, 336 departments confirmed the study was advertised either by email or as a poster. This advertisement contained a link to an open-access screening website. Twitter and Facebook were also used to circulate the advertisement to young people who expressed an interest in the following terms: stress; worry; rumination; mental health; self-esteem; well-being; research; psychology; CBT; and online therapy. In addition, 3 organizations working with young people or in the field of mental health agreed to advertise the study.

A 2-step procedure identified eligible participants. In the first step, an open-access screening website with conditional automated feedback identified potential participants for further screening by screening in those with elevated RNT (>75th percentile) using shortened versions of the PSWQ (4 items, range 4 to 20, cut-off ≥12) and RRS (5 items, range 5 to 20, cut-off ≥10), as developed by Topper et al [48]. A conservative cut-off of 15 on PHQ-8 [49] excluded individuals likely to be experiencing a current MDE. Eligible participants provided contact details as consent to be contacted for further telephone screening.

**Figure 1:**
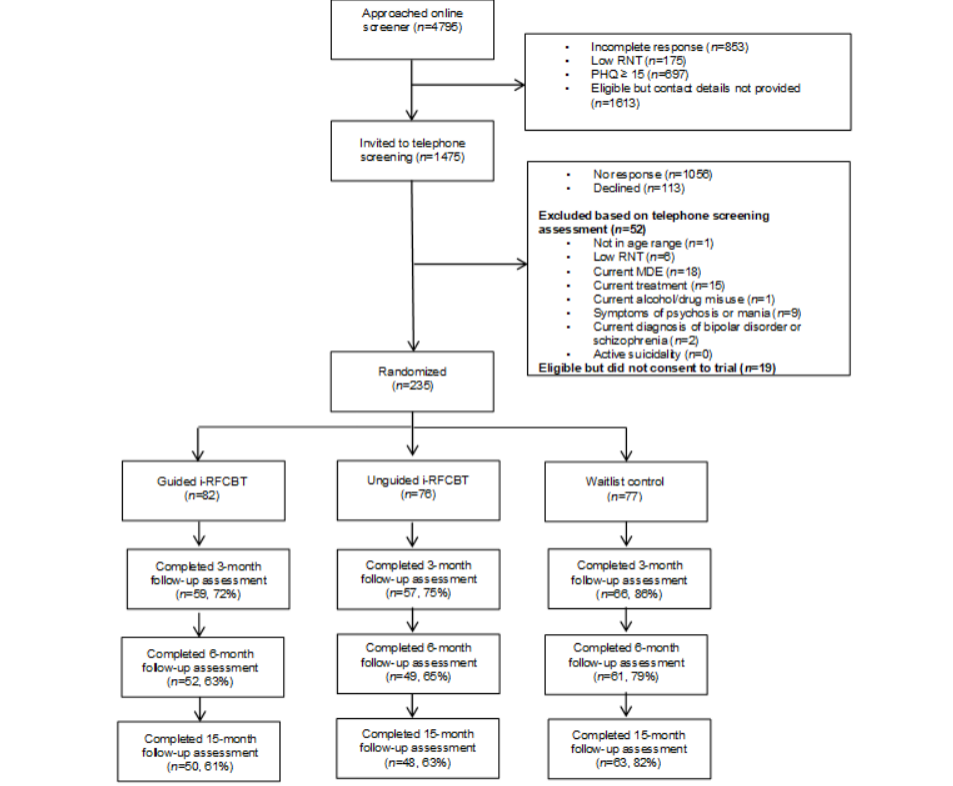
Consolidated Standards of Reporting Trials flowchart. MDE: major depressive episode; PHQ: Patient Health Questionnaire; RNT: repetitive negative thought; i-RFCBT: Web-based rumination-focused cognitive behavioral therapy.

In the second step, a telephone interview consisted of brief screening questions for alcohol and drug use, symptoms of bipolar disorder and psychosis (Psychosis Screening Questionnaire [PSQ]; [50]), assessment of any relevant past or current treatments, and the SCID-I [40] sections on current and past depressive episodes, dysthymia, and any relevant anxiety disorders and eating disorders. As the primary objective was to investigate the prevention of depression, diagnoses of anxiety disorders and eating disorders were recorded but participants meeting the criteria for any of these disorders were not excluded from the study. The consent to interview was obtained verbally and included providing their general practitioner’s (GP) contact details so that appropriate clinical support could be obtained in the event of disclosure of suicidal risk. The interview was audio-recorded, with consent, so that the diagnostic status could be independently checked. The same researcher conducted the baseline and follow-up telephone interview assessments, ensuring continuity of contact between the research team and participants. A total of 254 participants were eligible, of whom 235 returned written informed consent and were randomized to guided i-RFCBT (n=82), unguided i-RFCBT (n=76), or usual care control (n=77). The Consolidated Standards of Reporting Trials diagram (Figure 1) indicates the numbers excluded at baseline for each of the exclusion criteria.

#### Interventions

##### Guided Web-Based Rumination-Focused Cognitive Behavioral Therapy

The guided intervention was an English version of i-RFCBT (called MindReSolve), translated and adapted from the version used by Topper et al [27] to include case examples relevant to university students (see Multimedia Appendix 1). RFCBT differs from standard CBT by seeking to change the process of thinking rather than the content of individual thoughts [51]. RFCBT [52] was developed from theoretical models and experimental findings indicating distinct types of repetitive thought (RT) with different consequences [29]: unconstructive RT involves an abstract, evaluative processing mode focused on the meaning and evaluation of events and difficulties, leading to a range of negative consequences such as poorer problem-solving and greater emotional reactivity, relative to constructive RT, which involves concrete, specific, and action-oriented processing [53]. RFCBT therefore aims to shift participants from an abstract and evaluative style to a concrete, specific, and action-oriented style [29], consistent with evidence that concreteness training reduces depression [54].

RNT is also theoretically conceptualized as a mental habit acting as a form of avoidance and maintained by negative reinforcement [55]. RFCBT therefore involves counterconditioning the avoidant ruminative response with more helpful coping strategies and approach behaviors [52]. In practice, this involves the functional analysis of rumination to help users spot triggers for rumination, to distinguish between helpful and unhelpful RT, and to counter condition unhelpful RT with more functional responses through the formulation of contingency *If-Then* plans [52].

The internet treatment was delivered on the internet platform and software owned, programmed, and hosted by Minddistrict [56], accessed by a research licence purchased from Minddistrict by the research team. The specific content of the i-RFCBT intervention was developed and entered into the platform using its content management system by the research team led by Edward Watkins, using the same key intervention principles and techniques as face-to-face RFCBT as described by Watkins [52], adapted for the internet. i-RFCBT contains the same key components as face-to-face RFCBT [52], split into 6 1-hour modules, each in turn split into 3 or 4 sessions consisting of a single Web page, with 1 to 2 weeks recommended per module for practice of the techniques. The content includes psychoeducation, mood diaries, experiential audio exercises, pictures, and video vignettes of university students talking about their own experiences of the intervention. Modules follow the same basic structure: reflection on the previous module; introduction of a new technique; experiential in-session exercises; and plans for implementation. The specific behavior-change techniques are drawn from the following groups in the Behavior Change Technique (BCT) Taxonomy (v1) [57]: goals and planning (goal setting, action planning, review behavior, and behavioral contract), feedback and monitoring (self-monitoring of behavior and outcomes), shaping knowledge (information about antecedents), natural consequences (information about social and environmental consequences and monitoring of emotional consequences), associations (prompts/cues and associative learning), repetition and substitution (behavioral practice/rehearsal, behavior substitution, and habit formation), antecedents (restructuring the physical and social environment and avoiding/reducing cues for the behavior), and self-belief (mental rehearsal of successful performance, focus on past success, and self-talk). The key strategies include coaching participants to spot warning signs for rumination and worry, and then to make If-Then plans in which an alternative strategy is repeatedly practised in response to the warning signs. These strategies include being more active, slowing things down, breaking tasks down, opposite action, relaxation, concrete thinking, becoming absorbed, self-compassion, and assertiveness.

The intervention was accessed individually, for free, on a secure, password-protected website. Access was granted using an email link, inviting the participant to set up a personal account and password. The intervention was supported by qualified clinicians who had received additional specific training in RFCBT. This support consisted of asynchronous written feedback provided by the clinician at the end of each module. Feedback served to highlight positive steps and identified areas to focus on in the following module. Feedback was constrained by template responses for each module, faithful to the RFCBT model, which could be adapted to individual participants’ responses. All the content and module order were identical across participants, ensuring treatment fidelity. Each module was self-paced, but the participants were advised to spend 1 to 2 weeks on each and could only access the next module once feedback from the clinician was received, typically within 2 working days. Clinicians monitored log-ons and sent personalized reminder emails if there was no log-on for over a week. The platform also sent an automatic weekly reminder if the platform had not been accessed for a week. Suicidal risk was also monitored using a well-established departmental protocol to determine the level of risk and seek clinical support as appropriate.

Therapists were provided with regular supervision with the developer of RFCBT (EW) to further encourage treatment fidelity. All (100%) feedback reviewed by EW were faithful to the intervention model (over 10% of therapist feedback sampled—a minimum of the 3 initial feedback for each therapist, plus a random subset of later feedback).

##### Unguided Web-Based Rumination-Focused Cognitive Behavioral Therapy

The content of unguided i-RFCBT was almost identical to guided i-RFCBT with minor adaptations for self-help to include some automatic Web-based conditional feedback addressing common challenges with the exercises. Access was granted via an email link to set up a personal account and password. Participants could then access all modules without restriction but were advised to spend 1 to 2 weeks on each to allow time for practice. Responses were not monitored except for weekly checks of questionnaires to identify and follow up suicidal risk as necessary.

##### Usual Care Control Condition

Participants in the usual care control condition were permitted to access any other treatments during the study, as necessary. They were also offered access to unguided i-RFCBT at the end of the follow-up period.

#### Measures

All measures were completed at baseline, 3 months, 6 months, and 15 months unless otherwise stated. Diagnostic interviews for the primary outcome were conducted by telephone, with the option to complete self-report questionnaire measures for secondary outcomes during the telephone interview or request for them to be returned by email/post.

*The* *SCID-I* [40] is a semistructured diagnostic interview for Axis I DSM-IV diagnoses. The SCID-I was used to assess MDE (current and past), anxiety disorders, and eating disorders. The interrater reliability for Axis I diagnoses is fair to excellent, with a mean Kappa of 0.71 [58]. In the event of disclosure of suicidal risk during the diagnostic interview, the researcher followed a well-established departmental protocol to assess risk and obtain clinical support as needed.

*The Episodic Life Event Interview*, part of the University of California Los Angeles Life Stress Interview [59], assessed the number and impact of stressful events since the previous assessment (for the previous 3 months at baseline). Participants provided a list of events experienced and a subjective rating of stress experienced as a result of the worst event. The original scale ranges from 1 *none* to 5 *severe*. Participants scored 0 if no events were experienced. To aid analysis and interpretation, stress was recoded to collapse 0 *no event* and 1 *event experienced but no stress* into a single *no stress* category. The recoded stress scale therefore ranges from 0 *no stress* to 4 *severe stress*.

*The PSWQ* [46] is a 16-item self-report questionnaire assessing frequency, intensity, and automaticity of worry (eg, “My worries overwhelm me” and “I know I shouldn’t worry about things, but I just can’t help it”). It is scored from *1* (not at all typical of me) to *5* (very typical of me), with higher scores indicating higher levels of worry. The internal consistency is high with good test-retest reliability [46]. The PSWQ has also been shown to have good predictive validity for symptoms of anxiety and depression [60].

*The RRS* [47] is a self-report measure of frequency of ruminative responses to depressed mood, with items relating to the self (eg, “Think about all your shortcomings, failings, faults and mistakes”), one’s symptoms (eg, “Think about how hard it is to concentrate”) and possible causes and consequences of one’s mood (eg, “Go away by yourself and think about why you feel this way”). Items are scored from 1 (almost never) to 4 (almost always). Higher scores indicate higher levels of rumination. The RRS has good internal consistency, moderate test-retest reliability, acceptable convergent validity, and good predictive validity [47,61,62].

*The Patient Health Questionnaire* (PHQ-9; [63]) is a 9-symptom measure of depressive symptoms. Scores range from 0 to 27, with higher scores indicating greater severity. The PHQ-9 is a reliable and valid measure of severity of depressive symptoms [63].

*The GAD Screener* (GAD-7; [64]) is a standardized self-report measure of symptoms of anxiety. Scores range from 0 to 21, and higher scores indicate more severe symptoms. Spitzer et al [64] demonstrated good validity and reliability of the GAD-7.

##### Demographics and Treatment

At baseline, participants were asked if they had any family history of depression (including whom and how recently) and whether they had experienced any physical, sexual, or emotional abuse before the age of 16 years (yes/no questions with no further details requested). Participants were asked to report whether they had received any mental health treatments (medication, therapies, and use of self-help materials) before or during the trial. Timing, duration, and dosage (for medication) were recorded.

### Randomization, Allocation Concealment, and Blinding

Independent computer-generated block randomization (block size of 3), stratified by sex (male vs female) and a history of depression (presence or absence of past depressive episodes), was used to allocate participants to the guided i-RFCBT, unguided i-RFCBT, or usual care control in a 1:1:1 ratio. Varying block sizes were not used as the 2 levels of stratification ensured it would be difficult for the researcher to anticipate or determine allocation. A third party not involved in assessing or treating the participants implemented the random allocation sequence and informed the therapist of the condition for each participant. The researcher responsible for recruitment and screening was blind to allocation and unable to influence the order of consents. As a single blind trial, the researcher conducting outcome assessments was blind to allocation. The researcher was not involved with any element of treatment delivery. To preserve researcher blinding, participants were notified of their treatment allocation by a trial therapist. Owing to the nature of the intervention, participants and therapists could not be blinded.

### Statistical Analysis for the Phase III Efficacy Trial

Data cleaning followed the protocol set out by Tabachnick and Fidell [65]. Unplanned missing data were handled via multiple imputation (MI). A sensitivity analysis, assuming a variety of MI models (Missing at Random and Missing Not at Random), verified the likely impact of missing data. Auxiliary variables were used to improve the estimation of missing data. Primary analyses were conducted on the intention-to-treat (ITT) sample. Additional analyses assessed the effect of compliance using the Complier Average Causal Effect (CACE) analysis [66]. The CACE analysis provides an unbiased estimate of the benefits of compliance by comparing the compliers in the intervention group to a comparable subgroup of the control group who would have complied had they been offered the intervention. Compliance was defined in the protocol as fully completing at least 4 of 6 modules, that is, accessing all of the sections in each of those modules [45]. Analyses were carried out using statistical software, Stata (StataCorp; version 15.1 [67]).

As a prevention study, the primary outcome was the occurrence and time to onset of any depressive episode by 15-month follow-up. To investigate this, Cox proportional hazard models were fitted to the depression event data, with the diagnosis of an episode of major depression at any point during the follow-up period as the outcome and time to onset measured in weeks from the randomization date. Participants were censored upon measurement dropout or end of study. The Cox proportional hazard model was initially adjusted for both stratification variables: past depression and gender, as they have previously been found to influence likelihood of depression. In addition, as baseline stress was expected to increase the risk of depression and Topper et al [27] controlled for stressful life events, severity of baseline stress was included in the model. To examine the hypothesis that i-RFCBT would be especially beneficial in high ruminators experiencing high stress, we further tested the potential interactions between intervention condition and baseline stress, and intervention condition and history of depression within the Cox proportional hazard analysis.

Secondary outcomes of symptom severity and levels of rumination/worry were examined using mixed model analyses of covariance (ANCOVAs): between group (ITT/CACE) and repeated measures (3- to 15-month follow-ups), controlling for baseline symptom levels.

### Feasibility and Acceptability (Quasi-Phase II Pilot Arm)

Feasibility of data collection procedures was assessed by measuring the missing items on clinical outcome measures, number and timing of dropouts, and whether these varied across arms. The acceptability of the intervention was assessed using a behavioral index, measuring the number of Web-based modules completed.

### Ethical Approval and Informed Consent

Ethical and professional guidelines were followed at all times, in line with Good Clinical Practice guidelines. Ethical approval was obtained from the Ethics Committee of the School of Psychology, University of Exeter (Ref: 2012/554). Participants returned written informed consent including permission to contact their GP if significant risk was disclosed (see Multimedia Appendix 2).

## Results

### Demographics

For brevity, baseline demographics for the 3 arms are included in Table 1. As noted earlier, the primary comparison is guided i-RFCBT versus usual care control, with a separate analysis of the feasibility and acceptability of the adjunct unguided i-RFCBT arm.

### Survival Analysis: Guided Web-Based Rumination-Focused Cognitive Behavioral Therapy Versus Usual Care Control

A total of 27 participants in the primary comparison of guided i-RFCBT versus the control completed no follow-ups, and no minimum survival time could be estimated, so the ITT survival analyses were conducted on n=132 (guided n=63 and control n=69). Participants with a family history of depression were more likely to be lost to follow-up than those without: χ^2^_1_=3.89; *P*=.049. No other baseline variables were linked to loss to follow-up: all *t* values on continuous measures were <1.70; all chi-square test values on categorical variables were <1.74; all *P* values were >.09.

**Figure 2:**
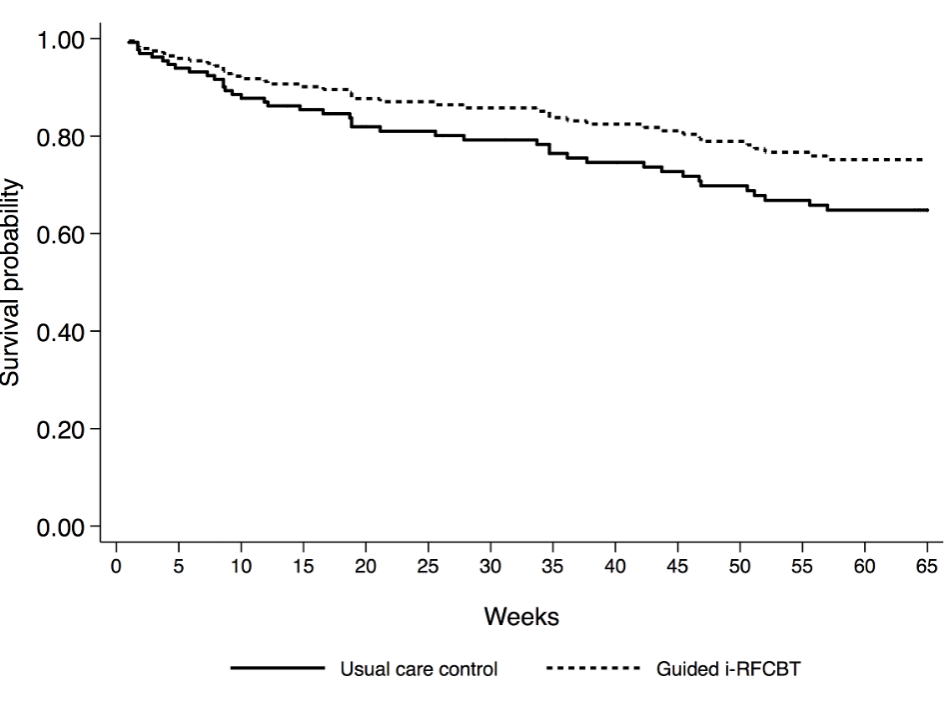
Survival curves for guided Web-based rumination-focused cognitive behavioral therapy (i-RFCBT) and usual care controls, adjusted for past depression and baseline stress.

There was no overall difference in incidence of depression (*P*=.64): 29% (n=18) of participants receiving guided i-RFCBT and 33% (n=23) of participants receiving usual care experienced an MDE during the follow-up period. A Cox proportional hazard model was conducted, including past depression, gender, and baseline stress as potential predictors of incidence of depression. As the majority (83%) of participants were female and there was no significant effect of gender in predicting depression, this variable was removed from the model, such that the final model controlled for past depression and baseline stress. As expected, history of depression significantly increased risk, with participants with a history of depression over two and a half times more likely to experience an MDE than participants without: hazard ratio (HR) 2.62, 95% CI 1.37 to 5.01; *P=*.004. Baseline stress marginally increased the risk of MDEs: HR 1.40, 95% CI 0.99 to 1.99; *P*=.06. When controlling for both past depression and baseline stress, there was a 34% reduced risk of depression in the guided i-RFCBT condition relative to usual care, although this difference was not significant: HR 0.66, 95% CI 0.35 to 1.25; *P*=.20 (see Figure 2).

For Cox proportional hazard models including the interactions between intervention condition and baseline stress, and intervention condition and history of depression, there was no differential effect of intervention between first onset (ie, no history of depression) or relapse/recurrence (ie, past history of depression) for incidence of major depression, and this interaction was removed from the final model (HR 0.54, 95% CI 0.15 to 1.94; *P*=.34; guided i-RFCBT: 38.9% first onset; 61.1% relapse vs usual care: 36.4% first onset; 63.6% relapse). Both the effects of past depression (HR 2.52, 95% CI 1.32 to 4.81; *P*=.005) and baseline stress (HR 1.99, 95% CI 1.22 to 3.24; *P*=.006) remained significant. As hypothesized, there was a significant interaction of the intervention condition by baseline stress (HR 0.43, 95% CI 0.21 to 0.87; *P*=.02), indicating a greater benefit of guided i-RFCBT relative to usual care (risk of MDEs decreased by 57%) for undergraduates with higher baseline stress.

**Table:** Baseline characteristics of usual care, guided Web-based rumination-focused cognitive behavioral therapy (i-RFCBT), and unguided i-RFCBT intention-to-treat samples.

Baseline characteristics	Usual care (n=77)	Guided i-RFCBT^a^ (n=82)	Unguided i-RFCBT (n=76)
Sex (female), n (%)	64 (83)	68 (83)	64 (84)
Age (years), mean (SD)	20.27 (1.55)	20.43 (1.65)	20.53 (1.30)
Ethnicity (white), n (%)	70 (91)	77 (94)	67 (88)
English mother tongue, n (%)	71 (92)	75 (91)	64 (84)
Previous major depressive episode (yes), n (%)	29 (38)	34 (41)	29 (38)
Received previous mental health treatment (yes), n (%)	38 (49)	38 (46)	31 (41)
Family history of depression (yes), n (%)	39 (51)	42 (51)	33 (43)
Parent with history of depression (yes), n (%)	34 (44)	34 (41)	29 (38)
Reported history of sexual abuse (yes), n (%)	7 (9)	5 (6)	5 (7)
Reported history of physical abuse (yes), n (%)	7 (9)	1 (1)	7 (9)
Reported history of emotional abuse (yes), n (%)	17 (22)	10 (12)	11 (14)
Patient Health Questionnaire-9, mean (SD)	5.6 (4.1)	5.6 (3.2)	5.4 (3.6)
Generalized Anxiety Disorder Screener-7, mean (SD)	6.6 (4.3)	7.3 (4.2)	7.1(4.0)
Penn State Worry Questionnaire, mean (SD)	61.9 (9.0)	62.0 (9.5)	60.3 (10.5)
Ruminative Response Scale, mean (SD)	47.9 (11.1)	49.8 (10.6)	47.2 (10.7)
Stressful events in the past 3 months, mean (SD)	3.6 (2.3)	3.8 (2.4)	3.4 (1.8)
Subjective rating of worst event, mean (SD)	2.20 (1.11)	2.57 (0.96)	2.53 (0.92)

^a^i-RFCBT: Web-based rumination-focused cognitive behavioral therapy.

**Figure 3:**
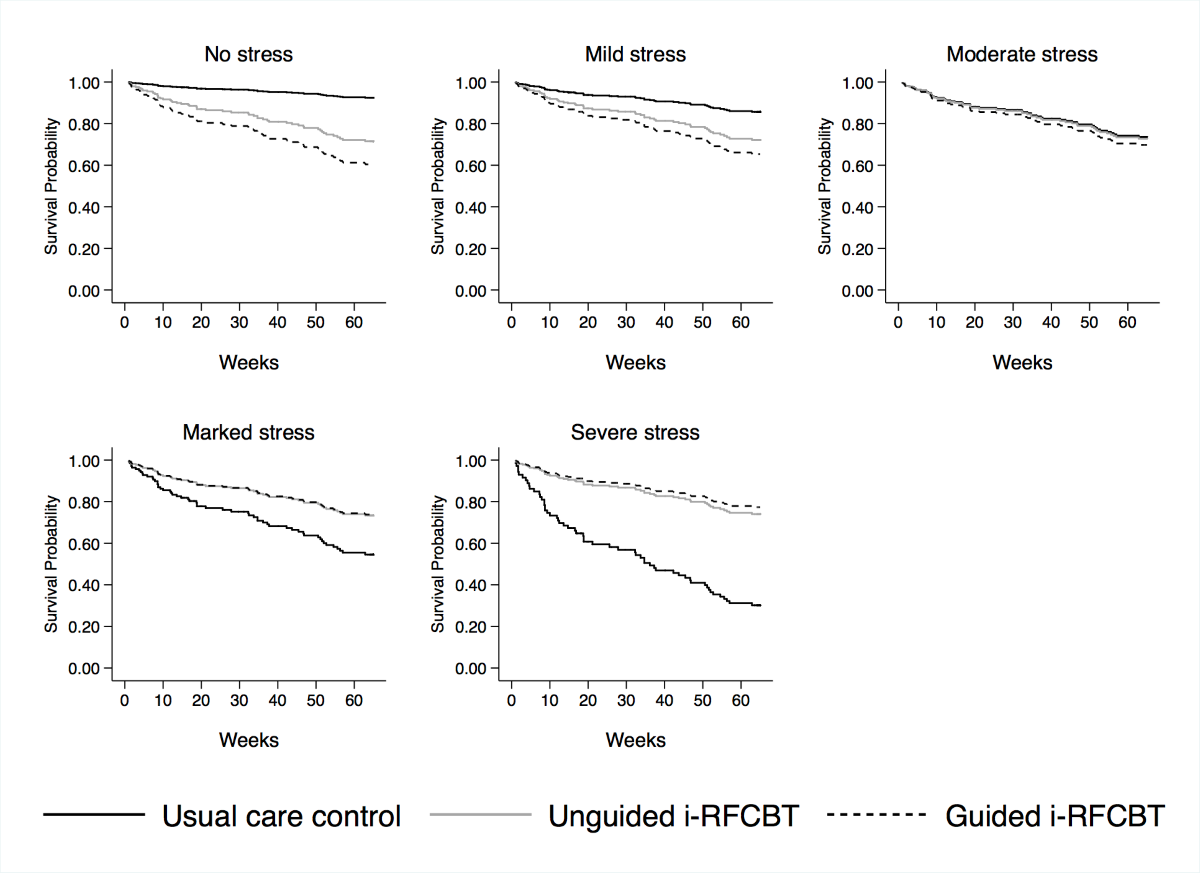
Survival curves for guided Web-based rumination-focused cognitive behavioral therapy (i-RFCBT), unguided i-RFCBT, and usual care controls at each level of baseline stress.

Plotting the interaction between the intervention group and baseline stress (see Figure 3) suggests that at higher levels of stress, guided i-RFCBT markedly reduces the risk of a depressive episode relative to usual care, with this effect reversing at low levels of stress (albeit in a small number of participants, only 13.1% scoring either 0 or 1).

As further sensitivity analyses, to investigate the effect of compliance on outcomes, we conducted a CACE analysis, using the Loeys and Goetghebur [66] method, which only allows for inclusion of the randomization variable in the model, and using regression-based adjustments to include past depression and baseline stress, which compares compliers in the intervention group to all other participants [68]. The mean completion for guided i-RFCBT was 3.46 (*SD* 2.25) for the full ITT sample (n=159), with 46% (38/82) being compliant by completing at least 4 of the 6 modules. The rates of compliance were higher among those with follow-up outcome data (n=132) as used for the CACE analysis at 60% (38/63). The results of the CACE analyses (see Multimedia Appendix 3) were equivalent to the ITT analysis. We therefore have only reported the primary ITT analysis.

### Secondary Analyses on Patient Health Questionnaire, Generalized Anxiety Disorder, Ruminative Response Scale, and Penn State Worry Questionnaire

Baseline adjusted ANCOVAs were conducted for each of the symptom measures, at each of the 3 follow-ups. Estimated means, between-group differences, and CIs are displayed in Table 2 for the case completers and following MIs (50 imputations). For the complete cases, at 3 months, rumination scores were significantly lower for guided i-RFCBT relative to usual care; at 6 months, both worry and depression scores were significantly lower for guided i-RFCBT relative to usual care, and there was no evidence of significant between-group differences at 15 months. Similar patterns were found when using MI to account for differing levels of missing data across the follow-ups.

**Table 2:** Baseline adjusted symptom measures at 3, 6, and 15 months: guided Web-based rumination-focused cognitive behavioral therapy versus usual care controls.

Timepoint and measure		Guided Web-based rumination-focused cognitive behavioral therapy, mean (95% CI)	Usual care, mean (95% CI)	Between-group difference, difference (95% CI)	Estimated between-group difference after multiple imputations, multiple imputation difference (95% CI)
**Follow-up 1 (3 months; n=114)**		
	PHQ-9^a^	4.75 (3.74 to 5.76)	5.40 (4.48 to 6.33)	−0.65^b^ (−2.02 to 0.72)	−0.55^b^ (−2.05 to 0.96)
	GAD-7^c^	5.58 (4.51 to 6.66)	6.27 (5.28 to 7.25)	−0.69^b^ (−2.15 to 0.78)	−0.53^b^ (−2.14 to 1.09)
	PSWQ^d^	57.27 (54.85 to 59.69)	58.45 (56.23 to 60.67)	−1.18^b^ (−4.46 to 2.11)	−0.75^b^ (−4.35 to 2.86)
	RRS^e^	44.34 (41.66 to 47.02)	48.21 (45.73 to 50.68)	−3.87^f^ (−7.53 to −0.21)	−3.69^b^ (−8.01 to 0.63)
**Follow-up 2 (6 months; n=105)**		
	PHQ-9	3.70 (2.48 to 4.92)	5.52 (4.42 to 6.62)	−1.82^f^ (−3.46 to −0.18)	−1.97^f^ (−3.87 to −.063)
	GAD-7	4.72 (3.44 to 5.99)	6.06 (4.91 to 7.20)	−1.34^b^ (−3.05 to 0.38)	−1.15^b^ (−3.16 to 0.85)
	PSWQ	54.83 (52.19 to 57.48)	58.41 (56.03 to 60.79)	−3.58^f^ (−7.14 to −0.02)	−2.71^b^ (−6.68 to 1.25)
	RRS	41.74 (38.15 to 45.34)	46.35 (43.12 to 49.58)	−4.60^b^ (−9.47 to 0.26)	−3.98^b^ (−9.48 to 1.52)
**Follow-up 3 (15 months; n=108)**		
	PHQ-9	4.47 (3.23 to 5.71)	4.82 (3.73 to 5.91)	−0.35^b^ (−2.00 to 1.30)	−0.38^b^ (−2.30 to 1.55)
	GAD-7	4.42 (3.16 to 5.68)	5.73 (4.62 to 6.83)	−1.31^b^ (−2.99 to 0.38)	−1.10^b^ (−2.10 to 0.80)
	PSWQ	54.81 (51.71 to 57.91)	58.11 (55.39 to 60.84)	−3.30^b^ (−7.43 to 0.82)	−1.74^b^ (−6.53 to 3.06)
	RRS	46.15 (42.59 to 49.72)	44.65 (41.53 to 47.78)	1.50^b^ (−3.28 to 6.28)	1.16^b^ (−3.99 to 6.31)

^a^PHQ-9: Patient Health Questionnaire.

^b^Not significant.

^c^GAD-7: Generalized Anxiety Disorder Screener.

^d^PSWQ: Penn State Worry Questionnaire.

^e^n=115 for Ruminative Response Scale (RRS) at 3 months owing to one partially completed questionnaire set.

^f^*P*<.05.

### Retention, Acceptability and Effect Sizes of Unguided Web-Based Rumination-Focused Cognitive Behavioral Therapy

A total of 19 (25%) unguided participants did not complete any follow-up assessments. Participants were significantly more likely to be lost to follow-up in unguided i-RFCBT than in usual care: χ^2^_1_=4.53; *P*=.03.

Owing to the exploratory nature of the unguided version of i-RFCBT, no formal CACE analysis of compliance was undertaken for the unguided intervention. In the full ITT sample, unguided participants completed an average of 2.66 modules (*SD* 2.35). The rates of compliance (38% unguided) were not significantly different from guided i-RFCBT (χ^2^_1_=1.08; *P*=.30). For the unguided intervention, participants logged in an average of 6.25 times (*SD* 5.21) and accessed the intervention over an average period of 114.92 days (*SD* 105.51). The median (interquartile range) was 87 days (22-173). Guided participants logged in an average of 7.97 times (*SD* 5.65) and accessed the intervention over an average period of 110.13 days (*SD* 108.44). The median (interquartile range) was 67 days (38-156).

### Estimates of Hazard Ratios for Unguided Web-Based Rumination-Focused Cognitive Behavioral Therapy Versus Usual Care

No formal significance analyses were undertaken, but hazard ratios and CIs were estimated relative to usual care. Using a Cox proportional hazard model including past depression and baseline stress, unguided i-RFCBT showed a 36% reduced risk of developing a depressive episode relative to controls: HR 0.64, 95% CI 0.33 to 1.24. A similar interaction between the intervention and baseline stress was found as for guided i-RFCBT (HR 0.48, 95% CI 0.23 to 1.00), such that unguided i-RFCBT had larger effect sizes for undergraduates with moderate-to-severe levels of baseline stress (see Figure 3).

**Table 3:** Baseline adjusted symptom measures at 3, 6, and 15 months: unguided Web-based rumination-focused cognitive behavioral therapy versus usual care controls.

Timepoint and measure		Unguided Web-based rumination-focused cognitive behavioral therapy, mean (95% CI)	Usual care, mean (95% CI)	Between-group difference, difference (95% CI)	Estimated between-group difference after multiple imputations, multiple imputation difference (95% CI)
**Follow-up 1 (3 months; n=116)**		
	PHQ-9^a^	4.02 (3.07 to 4.96)	5.21 (4.33 to 6.10)	−1.20 (−2.49 to 0.10)	−1.18 (−2.65 to 0.28)
	GAD-7^b^	4.94 (3.90 to 5.99)	5.98 (5.01 to 6.96)	−1.04 (−2.47 to 0.39)	−1.06 (−2.60 to 0.49)
	PSWQ^c^	55.77 (53.26 to 58.28)	57.60 (55.26 to 59.95)	−1.84 (−5.28 to 1.61)	−1.35 (−4.87 to 2.17)
	RRS^d^	44.47 (41.89 to 47.06)	47.01 (44.60 to 49.42)	−2.54 (−6.08 to 1.01)	−2.42 (−6.19 to 1.34)
**Follow-up 2 (6 months; n=104)**		
	PHQ-9	4.38 (3.20 to 5.56)	5.35 (4.30 to 6.40)	−0.97 (−2.56 to 0.61)	−1.04 (−2.89 to −0.81)
	GAD-7	4.20 (2.96 to 5.44)	5.93 (4.83 to 7.03)	−1.73 (−3.38 to −0.07)	−2.09 (−3.92 to −0.28)
	PSWQ	54.51 (51.60 to 57.42)	58.06 (55.47 to 60.65)	−3.55 (−7.46 to 0.36)	−3.35 (−7.36 to 0.67)
	RRS	41.27 (38.07 to 44.47)	45.20 (42.35 to 48.04)	−3.93 (−8.22 to 0.37)	−4.12 (−8.94 to 0.69)
**Follow-up 3 (15 months; n=107)**		
	PHQ-9	4.20 (3.00 to 5.40)	4.69 (3.64 to 5.73)	−0.49 (−2.08 to 1.11)	−0.92 (−2.61 to 0.77)
	GAD-7	4.49 (3.28 to 5.70)	5.52 (4.46 to 6.57)	−1.03 (−2.63 to 0.58)	−1.36 (−3.23 to 0.52)
	PSWQ	53.78 (50.75 to 56.81)	57.56 (54.93 to 60.19)	−3.78 (−7.79 to 0.24)	−4.34 (−8.57 to −0.09)
	RRS	42.07 (38.59 to 45.54)	43.85 (40.84 to 46.86)	−1.78 (−6.40 to 2.83)	−2.61 (−7.93 to 2.71)

^a^PHQ-9: Patient Health Questionnaire.

^b^GAD-7: Generalized Anxiety Disorder Screener.

^c^PSWQ: Penn State Worry Questionnaire.

^d^RRS: Ruminative Response Scale.

Between-group differences for unguided i-RFCBT versus usual care were estimated with baseline adjusted ANCOVAs for both case completers and using multiple imputations (50 imputations). Estimated means and CIs are displayed in Table 3. Owing to the exploratory nature of this comparison, significance testing was not conducted. Patterns of change and CIs indicate similar symptom changes to those found in the guided i-RFCBT versus usual care control ANCOVAs.

## Discussion

### Principal Findings and Comparison With Previous Work

The main aim was to test if guided i-RFCBT could be effective in preventing depression in undergraduate students in the United Kingdom over 1-year follow-up. When controlling for both past depression and baseline stress, guided i-RFCBT reduced the risk of experiencing an MDE by 34% relative to usual care. Although this effect size was not significant and smaller than that found by Topper et al [27], it is consistent with the wider prevention literature, which reports an average reduction in incidence of 21% [10] and a 28% (IRR=.72) reduction in incidence relative to controls for selective, predominantly CBT, interventions [11]. It may be that this study was underpowered to detect a main preventive effect of i-RFCBT as it used a larger effect size estimate derived from Topper et al [27].

As hypothesized, guided i-RFCBT was significant at preventing the onset of MDEs in high-risk undergraduates relative to usual care when they experienced moderate or above levels of baseline stress, with a hazard ratio of 0.43 when moderated by baseline stress. This is consistent with theoretical models of rumination and the RFCBT treatment approach. The tendency to ruminate about difficulties or low mood is more likely to increase the risk for depression in the context of stressful events, which activates that habitual tendency and provides subject matter to ruminate about. Even someone with a habitual tendency to ruminate is less likely to have frequent rumination in the absence of any difficulties. Furthermore, one key mechanism by which rumination is proposed to increase vulnerability to depression is by exacerbating and prolonging negative affect and distress [29,30]: rumination does not have deleterious effects in the absence of negative mood, and it is thus the confluence of stressful events that lower mood and the tendency to ruminate that particularly confer the risk for depression [32]. This pattern of results suggests a partial replication of Topper et al [27], by indicating that guided i-RFCBT may be a helpful preventive intervention for university students with high levels of rumination and worry, who also experience at least moderate levels of stress.

We note that the observed interaction between i-RFCBT and baseline stress could also be interpreted as indicating that RFCBT is unhelpful compared with usual care, for users who are experiencing little to no current stress. However, given the small number of participants who reported low levels of baseline stress, this reversal is based on low power and needs to be treated with caution.

The findings on the symptom measures suggest that guided i-RFCBT was effective in the short-to-medium term, by reducing rumination, worry, and symptoms of depression at 3 and 6 months relative to usual care, but that these improvements were not sustained over the longer term. Watkins and Nolen-Hoeksema [55] hypothesized that rumination could be conceptualized as a learnt habit, triggered by particular cues such as low mood. Within this analysis, successful long-term reduction of the ruminative habit requires extensive repetition and rehearsal of alternative more adaptive responses to the triggers for rumination. It may be that i-RFCBT was too brief or that participants did not practise enough to produce long-term change in the ruminative habit. It may also be that further engagement and booster sessions some months after the initial intervention phase would enhance the longer-term effects of the intervention [69]. These could take the form of explicit reminders to practice techniques (flashcards and text/email reminders; [70]) or increasing the generalizability of the new, more helpful techniques across a broader range of contexts [70].

One possible reason for the difference in findings between this study and Topper et al [27] is the means of assessing onset of depression: Reducing Stress and Preventing Depression (RESPOND) used structured clinical interviews, whereas Topper et al [27] used cut-offs on self-report questionnaires, which may overestimate incidence. Another potential explanation is the different samples. Although their sample included university students, the average age was 17.5 years, compared to 20.4 years in RESPOND. Cases of depression begin to rise steeply from the age of 14 [13], so it may be that the developmental risks during mid to late adolescence differ from those in university students and either that i-RFCBT was more efficacious in younger participants or the base rate was higher in the younger sample, increasing the power of the trial.

Compliance rates and the pattern of findings and preliminary effect sizes and CIs for unguided i-RFCBT were similar to those for guided i-RFCBT. These findings are in contrast to the literature on Web-based acute treatment for depression, which generally demonstrates larger effect sizes for guided interventions relative to unguided [41-43]. This benefit of therapist guidance has also previously been found for indicated preventive interventions in university students [71]. As a preliminary finding from a feasibility pilot, further large-scale trials are needed to confirm whether this potential equivalence between unguided and guided i-RFCBT is robust. We speculate that only selecting high-ruminating participants for the trial meant that i-RFCBT was highly relevant and engaging to participants, thus ameliorating the relative benefits of guidance on treatment motivation and completion.

Given the need for widespread dissemination of preventive interventions, an efficacious unguided intervention would be valuable, even if it had somewhat reduced effect sizes relative to the guided version, because it would not be constrained by therapist numbers or availability unlike a guided treatment and could be enormously scaled up to increase accessibility [44]. In addition, unguided interventions may benefit a previously unreached population as many university students do not seek professional help for mental health difficulties [22,23] and could therefore be more attracted to self-help interventions. In support of this, an unguided preventive intervention for students with elevated distress ratings reduced depressive symptoms relative to usual care at 2 months follow-up [72], with two-thirds of the trial completers reporting an unmet need (needing help but not seeking it) in the previous year. As i-RFCBT targets worry and rumination, rather than focusing on depression, this may further attract those who prefer self-help to manage their symptoms as worry is a common experience without the perceived stigma of mental illness [27]. These initial findings on the acceptability and effect sizes of the unguided version provide some promise in terms of potential benefits and suggests the value of further studies to formally test unguided i-RFCBT as a preventive intervention.

Despite the need for larger-scale trials to test the robustness of these findings, several strengths of RESPOND are identified. First, the RESPOND trial addressed some of the methodological limitations of the Topper et al [27] trial by including diagnostic interviewing. This allowed for retrospective diagnoses, capturing any episodes occurring between follow-up interviews, as well as baseline history of depression to assess the effect of previous history on risk of a further MDE.

The use of Web-based and telephone-based measures allowed for recruitment throughout the United Kingdom, with participants from a wide range of university departments and geographical locations, increasing the generalizability of the findings within this demographic. The target sample size was achieved through this recruitment strategy and this would therefore be a suitable approach for a larger scale trial of i-RFCBT.

### Limitations

There were several limitations to the study. First, the sample was disproportionately female, limiting the generalizability of the findings. However, females consistently report higher levels of rumination [73] and higher levels of depression, so a trial selecting on this basis will necessarily attract more female participants.

Second, despite a successful recruitment strategy, there was a considerable proportion of missing data at follow-up, particularly in the intervention conditions (albeit in the context of planning the sample size for 20% drop-out attrition). In addition, follow-up assessments were sometimes incomplete as participants did not always return the questionnaires after the follow-up interview, despite reminders being sent. Future trials should therefore further emphasize to participants the importance of follow-up data during the baseline assessment and ensure all measures are completed during the interview. Finally, common to many electronic mental health trials, the participants were not blind to the treatment condition, and, as such, the results could have been influenced by response bias and expectancy effects.

### Conclusions

Despite these limitations, taken together, the findings from the Topper et al [27] trial and from this RESPOND trial suggest that i-RFCBT is an effective and acceptable intervention for preventing depression in adolescents and undergraduates experiencing high levels of rumination and worry. This demonstrates the value in targeting a preventive intervention at identified risk factors. This intervention may be particularly effective in individuals experiencing high levels of stress. The initial findings relating to unguided i-RFCBT suggest this may be efficacious in preventing depression, which, if shown to be robust in a fully powered trial, would have significant implications for the scalability of i-RFCBT.

## Appendix

Multimedia Appendix 1 Screenshots of Web-based rumination-focused cognitive behavioral therapy.

Multimedia Appendix 2 Information sheet and consent form.

Multimedia Appendix 3 Complier average causal effect analysis.

Multimedia Appendix 4 CONSORT-EHEALTH checklist (V 1.6.1).

## Abbreviations

ANCOVA: analysis of covariance
CACE: complier average causal effect
CBT: cognitive behavioral therapy
CMD: common mental health disorder
GAD: generalized anxiety disorder
GP: general practitioner
HR: hazard ratio
i-RFCBT: Web-based rumination-focused cognitive behavioral therapy
IRR: incidence rate ratio
ITT: intention-to-treat
MDE: major depressive episode
MI: multiple imputation
PHQ: Patient Health Questionnaire
PSQ: Psychosis Screening Questionnaire
PSWQ: Penn State Worry Questionnaire
RCT: randomized controlled trial
RESPOND: Reducing Stress and Preventing Depression
RFCBT: rumination-focused cognitive behavioral therapy
RNT: repetitive negative thought
RRS: Ruminative Response Scale of the Response Styles Questionnaire
RT: repetitive thought
SCID-I: Structured Clinical Interview for DSM-IV
