# Supplementary material for: Reducing Stress and Preventing Depression (RESPOND): Randomized Controlled Trial of Web-Based Rumination-Focused Cognitive Behavioral Therapy for High-Ruminating University Students
Source: J Med Internet Res. 2019 May 13;21(5):e11349. doi: 10.2196/11349 (PMC6536298; doi:10.2196/11349)
Supplement: Multimedia Appendix 1 [file jmir_v21i5e11349_app1.pdf]

Home | 3 Tasks | Contact | Profile

## Module "MindReSolve"

Active | Waiting for feedback | Feedback available | Done | Pending | Cancelled

- Welcome
- Coping better with stress
- Introducing worry and rumination
- Unhelpful habits
- Mood Diary: Reflection
- Consequences of worry and rumination
- Causes and signs of stress and rumination

We'll look at ways to make you stronger. This way, stress and negative emotions don't knock you down the way they might when you're already feeling worn out or depressed.

We'll build up your strengths and the positive things in your life, so that stressful things won't pack as much of a punch, even when they do come up.

And by doing this you can improve your resilience and well-being.

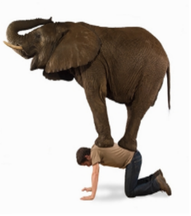

## Learning from your own experience of stress

One of the key things we will do throughout the training is to build and learn from your own experience and skills.

We will look closely at the times when you coped well with stress. We will also look closely at the times when you did not cope well with stress and see what we can learn by comparing the times you coped well with the times you coped less well.

We will see how you can build up good coping and reduce bad coping. Everyone has abilities and skills at coping. There are times when we all cope well. The trick is to do that more of the time.

## Exercise: Which situations do you find stressful?

We have made a list of three main categories where situations can be perceived as stressful:

1. Situations relating to school/college/work (including unemployment) (e.g., time pressure, too much work, not doing as well as hoped, not enough money, poor results)

2. Situations relating to family (especially parents, siblings and children)

3. Situations relating to friends and/or partners (e.g., boyfriend/girlfriend, including not being in a relationship)

Start the videos below to see which situations are stressful for John and Emma.

Now, practise using the Mood Diary, by completing one for today and yesterday combined.

- Across today and yesterday, think of a time during either day when you felt particularly **bad.** For example you could have been upset, depressed, nervous, or angry. Write down when this was and what the situation was. Describe which emotion you felt (in one or two words, and give a rating out of 10), how you responded (including whether you ruminated) and what the consequence of the way you responded was.
- Do the same for another time over the last 2 days when you felt **good, or a time when you felt a bit better**. Fill in when this was, what the situation was. Describe which emotion you felt (and give a rating out of 10), how you responded and what the consequence was.

| | Time Felt Worse | Time Felt Better |
|---|---|---|
| Write down what was going on when you felt that way.<br>When was this? Where were you, what were you doing, who was around? | | |
| Write down the emotion(s) you felt and rate on sliding scale from 0-10. You need to move the slider for the computer to register your response, so if you want to indicate 0, move the slider along a bit and then back to 0. | | |

# Learning to respond differently (and better) to stress

We can also start to make plans to do something different in response to the warning signs in order to stop the stress getting worse and to have a more effective response. These plans are a way to "*nip problems in the bud*" and to stop stress building up.

These plans only need to be simple plans of the following form - if I notice this sign of stress/ warning sign/ trigger, then I can do this more effective alternative. We call these plans **"If – Then" plans.**

For example, if feeling tense is a warning sign that you are about to worry, which it is for many people, then you could have a plan like "If I notice that I am getting tense, then I can do something relaxing to stop myself getting more wound up".

## Emma

Emma knows that she will feel bad when she is alone at home and doesn't have anything to do. Now, watch the next video in which Emma tells you how she could respond to this situation in order to feel better.

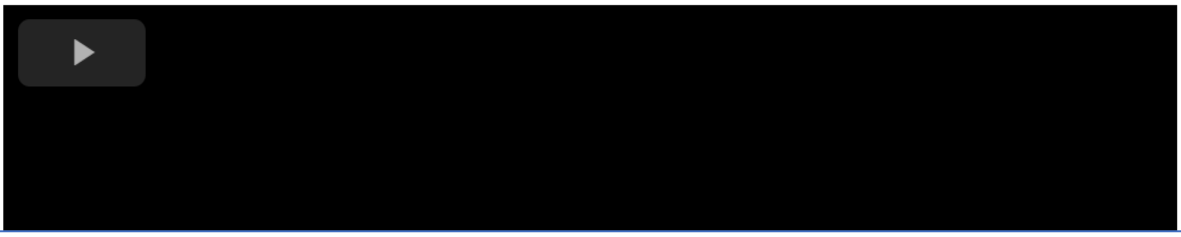

# Listening exercise

During the next listening exercise you are going to make a mental link between being faced with this problem and thinking it through in a concrete way. As best you can, try to keep the following plan in mind:

**When I**

notice my warning signs and stress signals,

**I will**

focus on being in the Present, so that I can come up with helpful HOW questions.

0:00

What was the effect of this exercise on your feelings, thoughts and bodily sensations?

Welcome to the fourth modu[illegible]line training! You've already finished half of the training. We've gone through a lot of information over the last few weeks. Hopefully you feel like you've learned something from it and it has helped you to start to feel more in control of your stress. This week you will be taught some new strategies and tools to use in stressful situations.

Before we move on, let's have a quick look at what you learned last week.

## Looking back at last week's module

Last week you practiced two new strategies.

First, you learned to **relax and focus on the present** when you notice stress and tension, so that you can calm down, reduce the tension, and make a clearer decision about what to do next.

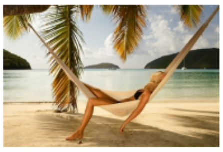

Second, you learned to think about your problems in a concrete and specific way by **asking yourself How-questions**. This concrete thinking helps to get problems into perspective and reduce overgeneralization, leads to better problem-solving and moves you toward action.

# How did it go with...?

Over the last week hopefully you practised being concrete and relaxing by repeatedly listening to the audio-recordings. You also continued to practise your IF-THEN plans in daily life.

Were you able to try out one or more of your IF-THEN plans? If so, which one(s)?

Over the last two weeks, how often have you been bothered by any of the following problems? Tick each relevant box.

| | Not at all | Several days | More than half the days | Nearly every day |
|---|---|---|---|---|
| Little interest or pleasure in doing things | ○ | ○ | ◉ | ○ |
| Feeling down, depressed, or hopeless | ○ | ○ | ◉ | ○ |
| Trouble falling or staying asleep or sleeping too much | ○ | ○ | ◉ | ○ |
| Feeling tired or having little energy | ○ | ○ | ◉ | ○ |
| Poor appetite or overeating | ○ | ○ | ◉ | ○ |
| Feeling bad about yourself –or that you are a failure or have let yourself or your family down | ○ | ○ | ◉ | ○ |
| Trouble concentrating on things, such as reading the newspaper or watching television | ○ | ○ | ◉ | ○ |
| Moving or speaking so slowly that other people could have noticed. Or the opposite –being so fidgety of restless that you have been moving around a lot more than usual | ○ | ○ | ◉ | ○ |
| Thoughts that you would be better off dead, or of hurting yourself in some way | ○ | ○ | ◉ | ○ |

Your responses on this questionnaire suggest you have been thinking about death or harming yourself. If you have had thoughts of suicide, we strongly recommend you contact your family doctor or general practitioner RIGHT AWAY for advice and tell them how you are feeling. If you don't think you can stay safe, please go to the nearest hospital accident and emergency room. If none of these options are available, please contact a family member or a trusted friend, so that you won't be alone right now. If your concerns are less urgent and you would find it helpful to talk confidentially about your feelings you can contact the Samaritans www.samaritans.org or www.befrienders.org.
