# Supplementary material for: Reducing Stress and Preventing Depression (RESPOND): Randomized Controlled Trial of Web-Based Rumination-Focused Cognitive Behavioral Therapy for High-Ruminating University Students
Source: J Med Internet Res. 2019 May 13;21(5):e11349. doi: 10.2196/11349 (PMC6536298; doi:10.2196/11349)
Supplement: Multimedia Appendix 2 [file jmir_v21i5e11349_app2.pdf]

Participant information sheet

Study Title: Targeting worry and rumination to prevent the onset of depression in young adults: A randomised-controlled trial comparing guided and unguided internet-based rumination focused cognitive behavioural therapy and a no treatment control.

This study is being conducted as a PhD project by Lorna Cook, supervised by Professor Edward Watkins, at the Mood Disorders Centre, University of Exeter. Please take a few minutes to read this information sheet and consent form carefully. Your participation is entirely your choice. Please take your time to think about whether or not you would like to participate. If you have any questions, please contact Lorna lzc204@exeter.ac.uk.

Why is this study being done? The purpose of the study is to investigate whether an internet-based therapy targeting worry and rumination is able to prevent depression in young adults. The therapy aims to help people to find better ways of handling day-to-day stress and reduce their risk of future problems, such as depression and anxiety. There are some positive early findings from the Netherlands to suggest that this therapy might be effective in preventing depression in teenagers and young adults. We are interested to find out if the same therapy might be effective in preventing depression in young adults in the UK.

As the therapy aims to target worry and rumination and prevent depression, it is important that participants have elevated levels of worry and rumination but are not currently depressed. You would still be eligible to take part if you have a history of depression, provided you are currently well.

What will happen if I agree to take part in the study?

- You will participate in a brief telephone interview to assess your eligibility, current and past symptoms of depression.
- If it is confirmed during the interview that you are eligible for the trial, you will be asked whether you consent to take part. You may give verbal consent immediately and return the consent form after the interview, or you can take some time to think about it and return the consent form if you decide to participate.
- The researcher will ask you to complete some baseline measures. You can choose to complete these during your telephone interview or ask for them to be sent to you to complete on paper/electronically. These questionnaires will assess symptoms of depression and anxiety. The baseline measures will also ask some brief questions about some risk factors for depression.
- To participate, you will need to provide us with a current email address, telephone number and your general practitioner's name and contact details.
- Once we have received your written consent, you will be allocated by chance to receive either internet-based rumination-focused CBT (RFCBT) guided by a therapist, a self-

help version of internet-based RFCBT or to a no treatment control group. There is a 2/3 chance of receiving some form of the therapy. However, we don't yet know if this therapy is effective in preventing depression, which is why the control group is so important.

- If you are allocated to the guided therapy group:
  - You will be allocated to a trained online therapist who will guide you through the internet therapy. They will give you feedback on the exercises and you can also send them messages.
  - You will be given a log-in and can work through the modules at a time that suits you.
  - The therapy consists of six modules and each module should take one to two weeks to complete.
  - You will be asked to complete some questions about your mood and depressive symptoms at the end of each module.
  - You will have access to the internet therapy for the whole length of the follow-up period so that you can continue to practise the techniques if you wish.
- If you are allocated to the unguided therapy group
  - You will have access to a self-help version of the internet therapy, which contains the same content but without the therapist contact.
  - This therapy will also consist of six modules and you can choose how quickly or slowly you complete them.
  - There will be no-one giving feedback on your responses while you are working through the therapy. However, a member of the team will monitor responses weekly in order to check for and follow up on any risk reported.
  - The programme will save your answers and the research team will view these as part of the data analysis.
- If you are allocated to the control group:
  - You will be asked to carry on as normal. If you feel the need, you will be allowed to access any other treatments of your choice throughout the study.
  - We will ask you at each follow-up to tell us what treatments, if any, you have used so that we can take these into account in our results.
- Follow-up measures:
  - All participants will be contacted at regular intervals (initially at 3 months from entering the study, and then 3 months and 1 year after that first follow-up) and asked to complete questionnaires about worry, rumination and symptoms of anxiety and depression. The same researcher who conducted your screening interview will also ask you to repeat that interview at each follow-up point. This longer term follow-up data is really valuable in helping us to understand if the internet therapy works or not.
  - With your permission, each of the telephone interviews will be audio-recorded to enable us to check the conduct and reliability of the interviews.

What side effects or risks can I expect from being in the study?

The risks of participating in the study are minimal. There are no known side effects of the therapy and controls will simply be asked to carry on as normal. However, given the nature of

the topic it is important to consider the following points before deciding whether or not to participate:

- Some of the questions ask about symptoms of depression or anxiety, including suicidal thoughts. You may find answering some of these questions makes you feel uncomfortable. If at any point you do not wish to answer a particular question you can simply refuse and move to the next question without giving any reason for doing so.
- If at any stage you are thinking about suicide, we urge you to speak to your GP or go to the nearest Accident and Emergency or phone one of the suicide hotlines at www.samaritans.org or www.befrienders.org
- If you report any suicidal thoughts on the research measures, the researcher will ask you some more detailed questions about your thoughts and feelings. The researcher is not a clinician and will follow a well-established procedure for assessing and acting on suicide risk to ensure you get appropriate clinical support. It may be necessary for the researcher to contact your GP directly to inform them of your difficulties. Depending on your responses, this would involve either sending a letter to your GP to notify them of your thoughts or phoning your GP to suggest they meet with you. In urgent cases, your GP or an ambulance would be contacted while you were still on the phone, in order to obtain immediate clinical help.
- For those in both the guided and unguided therapy, your responses on the questionnaires at the end of each module will be closely monitored and any suicidal thoughts you report will be followed up using the same procedure above to obtain appropriate clinical support for you. In the guided group, your therapist will see your responses when you submit a module and will follow up any risk as soon as possible. In the unguided group, as you are free to work through the modules at your own pace and we will not know when you have completed a particular module, a member of the team will log in on a weekly basis to check for any risk reported. Again, they will follow the procedure outlined above.
- In the control group, any suicidal thoughts will only be assessed during the follow-up measures.
- Your symptoms may not improve, or may get worse over the course of the study. We have good reason to believe that the therapy will help but we cannot guarantee that it will work for any one particular person.
- This is a research trial and we cannot provide treatment or support beyond what is included in the internet therapy. Your GP retains responsibility for your clinical care throughout the trial. If you are distressed by any of your symptoms, or the researchers tell you that your symptoms are worsening, you are advised to speak to your GP. They will be able to offer you support and advice about your mood and how to manage your stress better.

What are the benefits of taking part in the study?
The regular follow-ups will allow us to track your mood and notify you if any of your symptoms seem to be worsening. If your responses suggest that your mood requires attention, we may advise you to speak to your GP or a mental health practitioner.

We have evidence to suggest that the therapy may be helpful in reducing stress and risk of later depression or anxiety. You may therefore find that the therapy helps you to manage your problems more effectively.

If you are in the control group, your involvement in the trial will not take up much of your time. It will only involve completing the measures four times over the course of the study. During that time you will be able to access any other therapies you might find useful. Once you have completed the follow-ups you would be allowed to access the unguided version of the therapy if you so wished.

The information you provide may help us to better understand how to prevent depression in young adults. This will hopefully allow us to provide better therapies that help young adults manage their stress and worry and reduce their risk of depression.

Will my information be kept confidential?
Any information you provide will be kept strictly confidential, except where disclosure to third parties is required by law or the professional guidelines for psychologists. You will be asked to provide contact details for your GP so that we can contact them if there is any significant risk of harm to yourself or others.

You will be given a unique anonymity code so any information you provide will only be identifiable by the code, not your name. Any forms that do contain your personal details (such as the consent form) will be stored separately from the other materials. Your data will be stored securely at the University of Exeter using password protected files and lockable filing cabinets that can only be accessed by the research team.

What will happen with the results?
Results will be written up as part of Lorna's PhD thesis and potentially published in a journal. Your name or any other personally identifiable material will never be included in any written reports or publications. Any information presented at scientific meetings will also be fully anonymised. If you would like a copy of the findings once they are available, please email Lorna, and she will be happy to send you them to you.

Will I be paid for taking part in this study? You will not be paid for taking part. A regular prize draw will be held. At baseline and each time you complete a follow-up you will receive one entry ticket. You may choose to enter multiple tickets into one single draw or use them one at a time.

What will happen if I decide to participate? Can I change my mind?
Your participation in the study is entirely voluntary. You are free to choose whether or not to take part and if you choose not to, there will be no penalty to you in any way. If you decide to take part you will be asked to keep a copy of this information sheet and to sign the consent form. You have the right to withdraw at any point after signing the consent form, without giving a reason for doing so. Just send us an email if you wish to withdraw from the study.

Can I be stopped from participating?
The researchers may ask you to stop being in the study if they believe it is in your best interests, if you do not follow the study's rules or if the whole study is stopped.
Who can I contact if I have questions about the study?
If at any stage you have any questions, concerns or complaints about any aspect of the study, you can speak in confidence to one of the investigators: Lorna Cook or Professor Ed Watkins.

Questions or concerns about the study can also be addressed to Cris Burgess, Chair of the Ethics Committee, School of Psychology, University of Exeter.

Contact details

Lorna Cook (PhD student): lzc204@exeter.ac.uk

Professor Ed Watkins (Co-founder and Director of Mood Disorders Centre and Sir Henry Wellcome Building for Mood Disorders Research): E.R.Watkins@exeter.ac.uk

Dr Cris Burgess (Ethics Chair): C.N.W.Burgess@exeter.ac.uk

Thank you for reading this information sheet

Consent Form

Study Title: Targeting worry and rumination to prevent the onset of depression in young adults: A randomised-controlled trial comparing guided and unguided internet-based rumination focused cognitive behavioural therapy and a no treatment control.

Researchers: Lorna Cook, PhD student, supervised by Prof. Ed Watkins

Please write/type your initials in the boxes below if you agree with the following statements:

1. I have read and understood the information sheet provided and have had the opportunity to ask further questions.	
2. I understand that my GP retains clinical responsibility for me throughout the study and will be contacted in the event of any significant risk of harm to myself or others.	
3. I understand that my participation in the study is voluntary and that I may withdraw from the study at any time, without giving any reason for doing so.	
4. I give consent for my telephone interviews to be audio-recorded so that the research team can check the conduct and reliability of these interviews. (Note: You may still participate without consenting to this audio-recording)	
5. I give my informed consent to participate in this study.	

Participant Name (please print):

Participant email address:

Participant telephone number:

Participant signature: Date:

Researcher Name: Date:

Researcher Signature:

If you are willing to be contacted about participating in further research at the Mood Disorders Centre, please write your initials in the box below. If you do not wish to be contacted, simply leave this box blank.

I am willing to be contacted about participating in further research at the Mood Disorders Centre.	

Please return the completed consent form to Lorna Cook by email (lzc204@exeter.ac.uk) or by post: Sir Henry Wellcome Building for Mood Disorders Research, University of Exeter, Perry Road, EX4 4QG

You will receive a copy signed by the researcher for your records.
