# Supplementary material for: Reducing Stress and Preventing Depression (RESPOND): Randomized Controlled Trial of Web-Based Rumination-Focused Cognitive Behavioral Therapy for High-Ruminating University Students
Source: J Med Internet Res. 2019 May 13;21(5):e11349. doi: 10.2196/11349 (PMC6536298; doi:10.2196/11349)
Supplement: Multimedia Appendix 3 [file jmir_v21i5e11349_app3.pdf]

Supplementary file 1: Complier analyses for guided i-RFCBT vs. controls

By the Loeys and Goethghebur (2003) method, compliers in guided i-RFCBT did not have reduced incidence of depression relative to estimated compliers in usual care (HR: 0.81, 95% CI [0.33, 2.53], *P* = .69). Similarly, when regression-based adjustments for past depression and baseline stress were included, compliers in guided i-RFCBT did not have significantly reduced incidence of depression relative to non-compliers (HR: 0.58, 95% CI [0.22, 1.50], *P* = .26). However, compliance interacted with baseline stress, so that compliers for guided i-RFBCT had reduced incidence relative to non-compliers at higher levels of stress (HR: 0.35, 95% CI [0.16, 0.75], *P* = .01).
